# The effect of an orally-dosed *Caralluma Fimbriata* extract on appetite control and body composition in overweight adults

**DOI:** 10.1038/s41598-021-86108-2

**Published:** 2021-03-24

**Authors:** Amanda Rao, David Briskey, Carla dos Reis, Alistair R. Mallard

**Affiliations:** 1 RDC Clinical, Brisbane, 4006 Australia; 2 School of Medicine, University of Sydney, Sydney, Australia; 3 Faculty of Medicine, University of Queensland, Brisbane, Australia

**Keywords:** Biochemistry, Plant sciences, Biomarkers, Weight management

## Abstract

To examine the effect of a *Caralluma Fimbriata* extract (CFE) on biomarkers of satiety and body composition in overweight adults. A double-blind, randomised, placebo controlled trial to examine the effect of a Caralluma Fimbriata extract (CFE) on biomarkers of satiety and body composition in overweight adults. Eighty-three men and women aged between 20 and 50 years of age completed 16 weeks of daily supplementation with either CFE or placebo. Plasma cardiometabolic (lipid profile, glucose, insulin) and satiety (ghrelin, leptin, neuropeptideY) biomarkers, body composition, diet history and gastrointenstinal function were assessed at baseline, weeks 4, 8, 12 and 16. Subjects in the CFE and placebo groups were well matched and predominatly female 93% and 87.5%, with a mean age of 40.9 ± 6.7 and 39.5 ± 7.5 years and body mass index (BMI) of 30.0 ± 3.1 and 30.2 ± 2.9 kg/m^2^ respectively. There was a significant difference in plasma leptin concentration change between groups at week 16 (*p* = 0.04), with the placebo group increasing concentration (2.27 ± 4.80 ng/mL) while the CFE group (0.05 ± 4.69 ng/mL) remained the same. At week 16, the CFE group had significantly reduced their calorie intake from baseline compared to the placebo group (245 cal vs 15.8 cal respectively *p* < 0.01). The CFE group also had a significant reduction in waist circumference of 2.7 cm compared to an increase of 0.3 cm in the placebo group (*p* = 0.02). A weight increase from baseline was seen in the placebo group that was not observed in the CFE group (1.33 kg weight gain vs 0.37 kg weight loss respectively; *p* = 0.03). The placebo group also had a significant increase in fat mass, android fat mass, BMI and leptin compared to the CFE group (*p* = 0.04, 0.02, < 0.01 respectively). CFE was effective at maintaining bodyweight during a non-calorie controlled diet compared to a placebo. The mechanism responsible for this action is requiring further research and could be due to an increase in satiety receptor sensitivity.

## Introduction

Obesity now represents a significant health, societal, and economic concern for not only Australia but the world^1,2^. Once considered to be an issue only in high-income countries, the incidence of obesity is rising in middle and low-income nations^3^. In 2017, an additional 1 in 10 Australian adults were obese compared to 1995^1^. If this upward trend continues 40% of Australians will be classified as obese in the next decade^4^.

Despite the growing health concern of obesity, widespread adoption of lifestyle, nutritional and exercise interventions have been largely ineffective^5^. As a result, dietary supplements have been adopted to help reduce/prevent accumulation of body fat^6^. A 2008 study found that of 1444 people who made a serious weight-loss attempt, 33.9% had reported using a weight loss supplement^7^.

One type of supplement gaining popularity for weight loss are natural bioactive supplements (Functional foods). One such plant that shows promise is *Caralluma Fimbriata (C. Fimbriata)*, an edible succulent plant from the *Asclepiadaecea* family which is native to India, Pakistan and Afghanistan^8–11^. Plants of the *Asclepiadaceae* family, including *C. Fimbriata*, contain an array of constituents known for their appetite-suppressing effects in the hypothalamus, including pregnane glycosides^9,12–14^. Previous studies have proposed that the mechanism for appetite suppression by *C. Fimbriata* extract (CFE) is through down-regulation of ghrelin synthesis in the stomach and neuropeptide Y (NPY) in the hypothalamus, but the exact mechanism of action is unknown^15–17^.

Research examining the appetite suppressing and weight loss effects of CFE in both human and animal subjects has been positive^10^. However, to date only a few human trials have been conducted, with limited understanding of mechanism, therefore more research on humans is required. A study by^18^ demonstrated the efficacy of CFE to prevent body weight gain and positively alter lipid profiles in obesity-induced rats fed a cafeteria diet. Similarly^19^, found feeding rats a cafeteria diet and CFE for 90 days induced significant dose-dependent inhibition of food intake. In addition, dose-related observations were made for the prevention of body, liver and fat pad mass accumulation and beneficial alterations in serum lipids associated with obesity. Furthermore, CFE has the potential to inhibit hyperplastic obesity in-vitro^19^. Preliminary human research has shown CFE dosed at one gram daily for 16 weeks, in conjunction with a hypocaloric diet, can reduce central adiposity in overweight adults compared to a placebo^8^. Another study reported similar results where one gram of CFE taken daily for 60 days, successfully suppressed appetite and reduced waist circumference in overweight adults^16^. However, a recent study following a similar protocol (1 g/day, 12 weeks) failed to show efficacy of CFE on appetite control and anthropometry measures in overweight and obese adults^20^.

Therefore, this study aimed to assess the efficacy of CFE on appetite control and body composition in overweight males and females to provide further clarity on its effectiveness and mechanism of action. It is hypothesised, that supplementation of CFE for 16 weeks would positively alter biomarkers of satiety and reduce overall caloric intake, resulting in body weight reduction compared to a placebo.

## Methods

**Figure 1:**
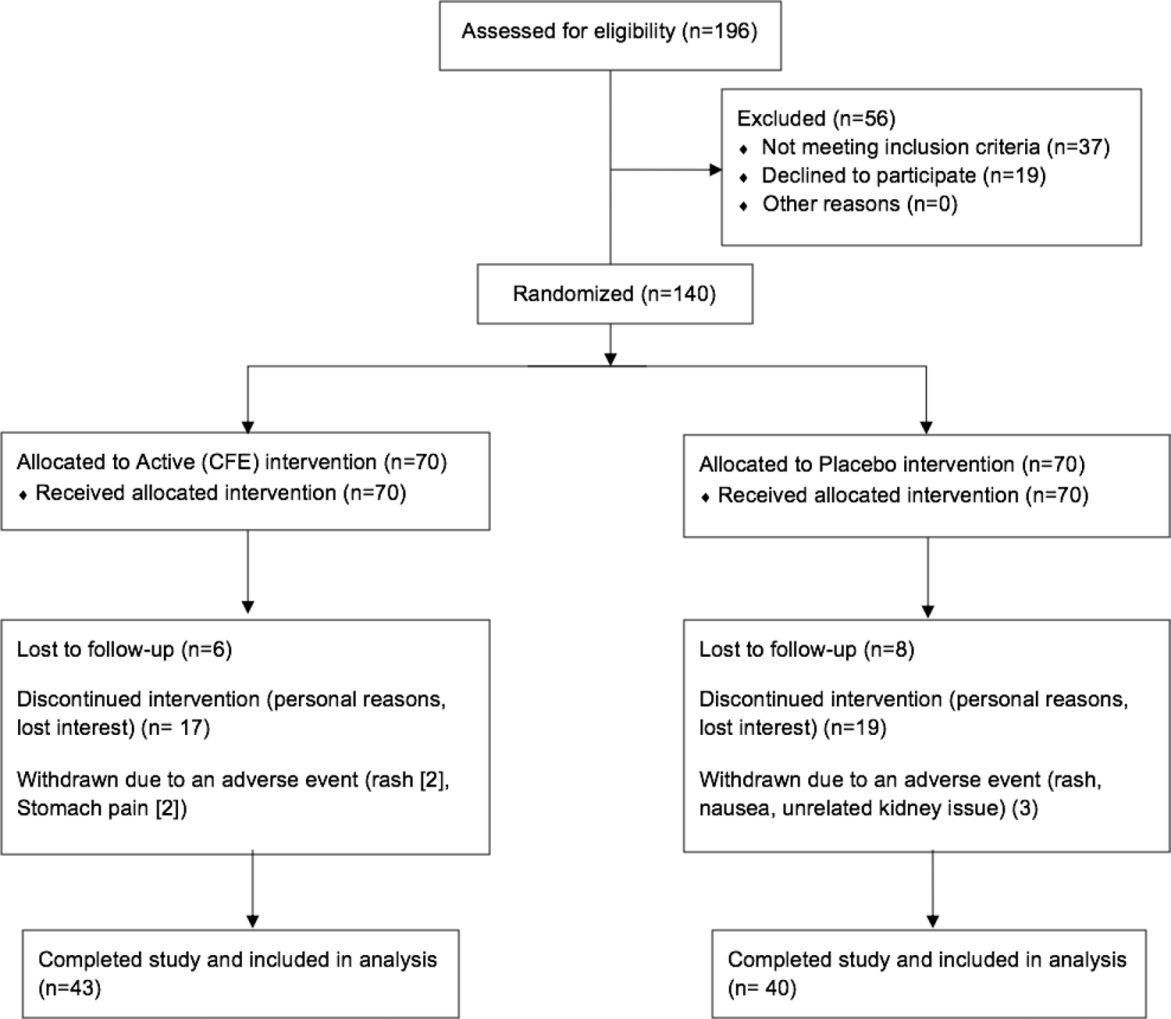
Participant flow diagram.

This double-blind, randomised, placebo controlled study recruited 140 overweight and obese (BMI 25–34.9 kg/m^2^), male and female volunteers, between 20 and 50 years of age who provided written informed consent. Of the 140 recruited participants, 83 completed the trial and were included in the final analysis (Fig. 1). This trial was conducted in Brisbane, Australia, between April 2018 and March 2019 in compliance with the current International Conference on Harmonization (ICH) Guideline for Good Clinical Practice (GCP), the Therapeutic Goods Administration (TGA) Note for Guidance on Good Clinical Practice, and ethical guidelines outlined in Additional Ethical Considerations. It was approved by Bellberry Limited Human Research and Ethics committee (Approval Number—201611834) and registered on the Australian New Zealand registry of clinical trials (ACTRN12617000872336).

In addition to the age and BMI requirements, male and female participants were excluded from the study if they reported any significant or unstable medical conditions including, but not limited to, cardiovascular, neurological, psychiatric, renal, gastrointestinal, immunological, endocrine or haematological abnormalities. Other exclusion criteria included undergoing treatment for cancer in the past two years, overnight shift workers, alcohol abuse, consumption of four or more caffeinated beverages daily, currently taking supplements or functional foods targeted at weight-loss, smokers and recreational drug users. Females who were pregnant, attempting conception, lactating, or not using contraception, were also not eligible for study participation. Participants were screened for known allergies or adverse reactions to the interventional product and the placebo; however, none were reported.

After providing informed consent, eligible participants undertook a health assessment and provided information on their current medication usage and medical history. Anthropometric measurements including body weight, height, waist and hip circumference, blood pressure, and heart rate were recorded, and a fasting (12 h) blood sample was drawn from an antecubital vein to an ethylenediaminetetraacetic acid (EDTA) vacutainer. Body mass index (BMI) was calculated using the measurements recorded for height and weight [BMI = weight (kg)/height (m)^2^]. Participant’s energy/caloric intake was assessed using a three-day food diary (Foodzone, AUSNUT 2011–13 and NUTTAB 2010 Sydney, Australia). Supplementary to the three-day food diary participants also completed a 24- hour food recall and food frequency questionnaire. Subjective satiety was assessed with a motivation to eat satiety visual analog scale (VAS). Participants rating of fatigue was measured using the visual analog scale to evaluate fatigue severity (VAS-F) questionnaire. A body composition scan (dual-energy X-ray absorptiometry, DXA) was performed to determine total mass, fat mass, fat-free mass, android fat mass and gynoid fat mass (Hologic Discovery, Massachusetts, USA). Android fat was assessed for the area of the abdomen from a line joining the two superior iliac crests and extended cranially for 20% of the distance between to the base of the skull. Gynoid fat was assessed for the portion of the legs from the greater femoral trochanter, extending caudally to mid-thigh. All repeat measures were conducted by qualified (minimum of public health degree) investigators at approximately the same time of day (within an hour of previous visits), with the same measurement tools and using standardised procedures and conditions (e.g. fasted, no exercise).

Following recruitment, participants were allocated to either the placebo group (n = 70) or the CFE group (n = 70) using the random allocation software, Sealed Envelope (sealedenvelope.com, London, United Kingdom). Blinding of the participants and investigators was maintained throughout the study. The CFE extract (Slimaluma) was provided by Gencor Pacific Limited (Lantau Island, Hong Kong) as a dry extract concentrate of *Caralluma Fimbriata* (aerial parts). The final product was produced by BlueGum Pharmaceuticals (Sydney, Australia). Both CFE and the placebo product were housed in opaque gelatin capsules to appear identical to one another. The participants randomised to the CFE group received capsules containing 500 mg of CFE, whereas participants randomised to the placebo product received capsules containing 500 mg of maltodextrin; a polysaccharide commonly used as a food additive. Participants were instructed to take one capsule before breakfast and one capsule before dinner with water for the full 16 weeks*.* This regime was selected on the basis of current standard dosing guidelines for the investigational product on the market.

Participants were asked to maintain their current diet and exercise regime but were not placed on a calorie-controlled diet or exercise program.

Participants attended the study site at weeks 0, 4, 8, 12 and 16 for assessment of their weight, waist and hip circumference, satiety (VAS), fatigue (VAS-F), dietary intake (24-h food and drink recall and FFQ used to validate the diet diary data), and product tolerance (gastrointestinal tolerance questionnaire, GIT-Q). Participants were monitored for compliance with the protocol and change of diet and exercise throughout the trial by a combination of telephone, fortnightly online diaries, and email communications, in addition to each scheduled site visit. At week 16, participants also had further fasted blood samples taken, DXA and 3-day food diary recorded. Any adverse events, either reported by the participant or observed by the trial supervisor, were recorded.

Participants recorded diet data at baseline, week 4, 8, 12 and 16 of the trial with data recorded over 3 days (2 weekdays and 1 weekend day). Participants were instructed on how to complete the diary and provided information on estimating portion sizes. Caloric and macronutrients intake from recorded diet data was calculated using Foodzone.

Collected blood samples were immediately centrifuged at 2500 rpm for 10 min at 4 °C. EDTA plasma was aliquoted and stored at − 80 °C until biochemical assays were performed.

### Biochemical analysis

Plasma leptin and ghrelin (primary outcomes) were analysed on a Luminex 200 using kits and calibrators from (Milliplex Human Metabolic Panel Cat#HMHEMAG-34K). Plasma serotonin, NPY, agouti-related peptide (AgRP), and cholecystokinin (CCK) were all analysed on a Sunostik plate reader as per kit instructions from (IBL ELISA kit Cat# IBRE59121, ELISA kit Cat#EKE04903, ELISA kit Cat# EK-003–53, ELISA kit Cat# EKE06904). Plasma IGF-1 and cortisol were analysed using a Diasorin XL Liaison using kits and calibrators from the manufacturer (Kit Cat# 313231). Product safety was assessed via plasma E/LFT (including lipid profile, glucose and insulin) with a Biobase BK400 analyser and associated Biobase reagents.

### Statistical Analysis

All data analyses were completed using GraphPad Prism (version 8.3.0 for Windows, La Jolla, California, USA). A sample size of 43 per group was calculated (G*power, version 3.1.9.4, Dusseldorf, Germany) based on the power to detect a change of 30% in plasma ghrelin concentration. Effect size: 0.7, alpha error probability: 0.05, power 0.9. Normality testing of the data was performed using the Shapiro–Wilk test. Outliers were tested using a Grubbs test. Baseline participant characteristics between groups were analysed using an independent samples t-test. Biochemical and body composition data between groups was analysed using an independent samples t-test for each time point as well as change. In addition hormone data and fat mass was calculated as individual change at week 16 (∆0–16) and analysed via t-test. Repeated measures ANOVA tests were used to analyse the effect of “time” (baseline, week 4, week 8, week 12, and week 16) and “group” (CFE vs. Placebo) with a Greenhouse–Geisser correction where appropriate. Planned pairwise comparisons were made with repeated measures t-tests and the Bonferroni post-hoc adjustment. Correlations were analysed using a Pearson r test. Statistical significance was set at *p* < 0.05. Data are presented as mean ± SD.

### Ethics approval

This trial was conducted in compliance with the current International Conference on Harmonization (ICH) Guideline for Good Clinical Practice (GCP), the Therapeutic Goods Administration (TGA) Note for Guidance on Good Clinical Practice, and ethical guidelines outlined in Additional Ethical Considerations. It was approved by Bellberry Limited Human Research and Ethics committee (Approval Number—201611834).

### Consent to participate

Participants provided written informed consent.

### Consent for publication

The authors have consent to publish all data.

## Results

Eighty-three participants completed the study (43 CFE, 40 placebo; Fig. 1). There were no significant differences in demographic measurements between groups at baseline (Table 1). No outliers were identified (± 2 SD of the mean) at any visit for any measure.

**Table 1:** Baseline participant demographics.

	CFE (n = 43)	Placebo (n = 40)
Female (n)	40	35
Male (n)	3	5
Age (years)	40.9 ± 6.7	39.5 ± 7.5
Weight (kg)	81.7 ± 9.4	85.2 ± 11.7
Height (cm)	165.2 ± 5.8	167.7 ± 8.4
Body mass index (kg/m^2^)	30.0 ± 3.1	30.2 ± 2.9
Waist circumference (cm)	94.8 ± 8.6	96.1 ± 8.8
Hip circumference (cm)	112.6 ± 6.5	113.4 ± 7.2
Systolic Blood pressure (mmHg)	120.8 ± 17.2	126.8 ± 18.3
Diastolic Blood pressure (mmHg)	80.6 ± 13.7	83.9 ± 10.5
Resting heart rate (bpm)	72.4 ± 10.7	67.6 ± 9.8

Data presented as mean ± SD; CFE, *Caralluma fimbriata*; bpm, beats per minute.

**Figure 2:**
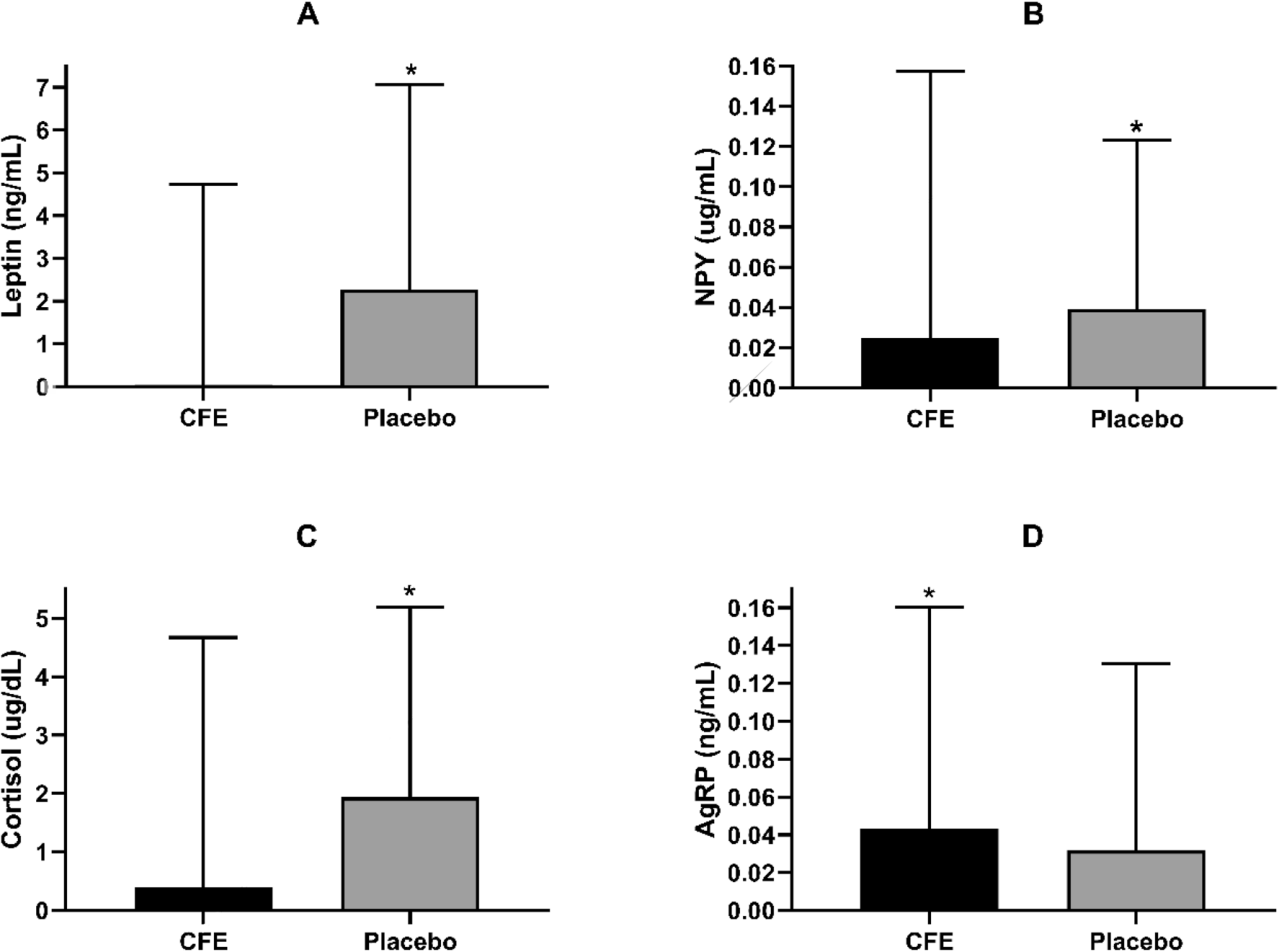
Change from baseline to week 16 for CFE and placebo groups. (**A**) plasma leptin concentration; (**B**) NPY concentration; (**C**) cortisol concentration; (**D**) AgRP concentration; Data presented as mean ± SD; CFE, *Caralluma Fimbriata*; NPY, neuropeptide-Y; AgRP, agouti related peptide *significant difference between groups at *p* < 0.05.

Plasma leptin concentration significantly increased at week 16 in the placebo group compared to the CFE which remained constant (*p* < 0.05; Fig. 2). No differences were seen between the CFE and placebo groups for plasma ghrelin at baseline (1103 pg/mL ± 752, 898 pg/mL ± 616 respectively) or week 16 (1033 pg/mL ± 679, 856 pg/mL ± 544 respectively). Serotonin, CCK, IGF-1 or E/LFT concentrations also showed no significant changes. NPY and cortisol significantly increased from baseline in the placebo group only (*p* < 0.05; Fig. 2). AgRP significantly increased from baseline in the CFE group only (*p* < 0.05; Fig. 2). No significant difference between groups was seen for either satiety (VAS) or fatigue scores (VAS-F).

**Figure 3:**
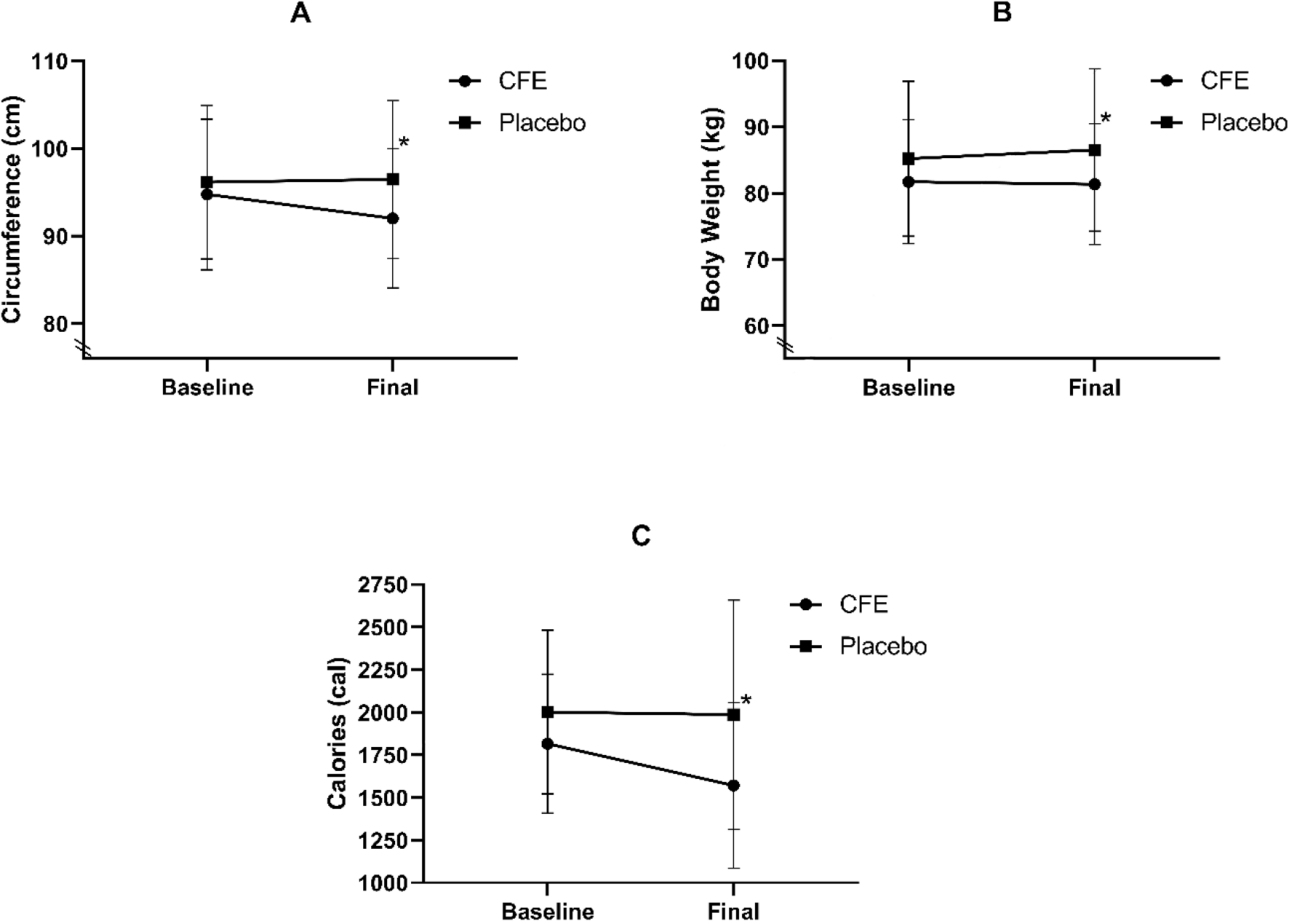
Morphology and caloric intake values at baseline and week 16 for CFE and placebo groups. (**A**) Waist circumference; (**B**) body weight; (**C**) energy/calorie intake; Data presented as mean ± SD; CFE, *Caralluma Fimbriata*; *significant difference between groups at *p* < 0.05.

Following 16 weeks of supplementation, the CFE group significantly reduced their caloric intake from baseline compared to the placebo group (245.0 cal vs 15.8 cal respectively; *p* < 0.01; Fig. 3). Diet diary data results was supported by the FFQ and 24-h food recall data. In addition, the CFE group also had a significant reduction in waist circumference of 2.7 cm compared to a gain of 0.3 cm in the placebo group (*p* = 0.02; Fig. 3). A weight increase from baseline was seen in the placebo group that was not observed in the CFE group (1.33 kg weight gain vs − 0.37 kg weight loss respectively; *p* = 0.03; Fig. 3). When individual change from baseline was calculated and averaged (∆ 0–16), the placebo group had a significant increase in fat mass, android fat mass and BMI compared to the CFE group (*p* = 0.04, 0.24 and < 0.01, respectively; Table 2).

**Table 2:** Morphology details from DXA scans over 16 weeks of supplementation.

	CFE (n = 43)			Placebo (n = 40)		
	Baseline	W16	Δ (0–16)	Baseline	W16	Δ (0–16)
Android (g)	2384 ± 725	2409 ± 783	26 ± 252	2440 ± 788	2,587 ± 855	147 ± 274*
Gynoid (g)	5439 ± 1237	5426 ± 1212	− 13 ± 337	5457 ± 1289	5557 ± 1396	99 ± 430
Fat mass (kg)	30.36 ± 5.56	30.48 ± 5.52	0.12 ± 1.88	30.82 ± 6.19	31.98 ± 6.88	1.12 ± 2.06*
Lean mass (kg)	49.82 ± 6.07	49.58 ± 5.91	− 0.24 ± 1.53	52.7 ± 8.32	53.05 ± 8.52	0.3 ± 1.67
BMI (kg/m^2^)	29.97 ± 3.09	29.82 ± 3.08	− 0.14 ± 0.68	30.22 ± 2.89	30.84 ± 3.17	0.62 ± 1.21*

Data presented as mean ± SD; CFE, *Caralluma fimbriata*; W16, week 16; Δ, change; BMI, body mass index;

*significant change between groups at *p* < 0.05.

When subgroup analysis was conducted for only female participants, the findings were the same as for the full cohort analysis. There were insufficient numbers to do a subgroup analysis on male participants.

When correlation analysis was conducted on study variables, a number of outcomes showed a significant correlation in the CFE or placebo group that was not seen in the other group (Table 3).

**Table 3:** Correlation of study variables for the CFE and placebo group.

CFE				Placebo			
Variable 1	Variable 2	*p* value	Pearsons r	Variable 1	Variable 2	*p* value	Pearsons r
Δ leptin	W16 IGF	0.044	− 0.324	W16 Leptin	Δ FM	0.002	0.492
Δ Cortisol	W16 WC	0.009	− 0.413	W16 Leptin	W16 Cortisol	0.008	− 0.43
Δ Cortisol	W16 WHR	0.015	− 0.386	W16 Leptin	Δ Cortisol	0.004	− 0.47
Δ Cortisol	Δ Ghrelin	0.001	− 0.523	W16 Cortisol	W16% LM	0.035	0.343
W16 Cortisol	Δ Calories	0.044	0.37	W16 NPY	W16 Weight	0.018	0.381
W16 Cortisol	W16 Serotonin	0.037	− 0.327	W16 NPY	Δ BM	0.024	0.365
Δ NPY	Δ AgRP	< 0.0001	0.782	W16 NPY	W16 Android Fat	< 0.0001	0.545
W16%FM	W16 Leptin	< 0.0001	0.532	W16 NPY	W16 Ghrelin	0.045	0.331

CFE, *Caralluma Fimbriata*; Δ, change from baseline values; W, week; FM, fat mass; WC, waist circumference; WHR, waist to hip ratio; LM, lean mass; NPY, neuropeptide-Y; BM, body mass; AgRP, agouti-related peptide.

CFE was well tolerated with normal E/LFT results and only 4 of the participants consuming CFE reporting mild side effects such as a rash, and minor gastrointestinal symptoms (bloating, loose stools) in the GIT-Q. An additional 3 of participants consuming the placebo product reported symptoms of nausea, rash and unrelated kidney problems on the GIT-Q (Fig. 1).

## Discussion

This study assessed the efficacy of CFE on appetite control and body composition in overweight males and females to provide further clarity on its effectiveness and mechanism of action. Primary outcomes included plasma leptin and ghrelin concentrations. Secondary outcomes included: biochemistry analysis (plasma NPY, cortisol, AgRP, serotonin, CCK, IGF-1, cortisol and E/LFT), satiety scores (VAS), reduction in overall energy/caloric intake, body composition changes (waist circumference, body weight, lean muscle mass, total abdominal fat, visceral fat, subcutaneous fat and android vs gynoid fat, BMI) and product safety/tolerance.

Of the primary outcomes, plasma ghrelin showed no significant difference between groups; however, there was a significant difference for plasma leptin. Several secondary outcomes also demonstrated beneficial effects of CFE. Following 16-weeks of supplementation, the CFE group maintained their baseline body mass, while the placebo group significantly increased body mass. Furthermore, the placebo group had an increase in android fat not seen in the CFE group. The maintenance of body mass in the CFE group coincided with a significant reduction in caloric intake not seen in the placebo group. The post prandial satiety score remained the same in both groups, despite a reduction in calorie intake in the CFE group. These results indicate that CFE supplementation was able to maintain body weight by most likely reducing calorie intake and therefore preventing an increase of android fat.

These results are consistent with existing literature in humans showing CFE is capable of altering body composition, satiety and waist and hip circumferences^8,16,21^. Kuriyan and colleagues^16^ found that with up to 60 days of supplementation with CFE, waist circumferences and hunger levels were reduced compared to a placebo group. Furthermore, there was a trend for CFE to reduce body weight, hip circumference and energy/caloric intake compared to the placebo group. Astell and colleagues^8^ showed 12 weeks of CFE supplementation reduced waist circumference compared to a placebo group and Griggs and colleagues^10^ showed that just 4 weeks of CFE supplementation reduced hyperphagia in a population with Prader-Willi syndrome. Previous research into CFE by Kamalakkannan and colleagues^22^ demonstrated adipocyte cell growth reduction in a CFE dose and duration dependent manner. Pre-adipocyte cell viability was reduced in cells treated with CFE through the inhibition of cyclin D1 transfer. This mechanism could have contributed to the observed prevention of android fat accumulation. Together, these results indicate the efficacy CFE supplementation can have on both body composition and satiety.

The clinical importance of these findings in 16-weeks is also to be considered. BMI has been shown to have a strong correlation with the incidence of cardiovascular diseases with Csige and colleagues showing that an increase in BMI by 1 kg/m^2^ increases the risk of heart failure by as much as 7% in females and 5% in males^23^. With BMI considered, based on the findings of Csige and colleagues, in this 16-week study, participants in the CFE group could have maintained their baseline risk factor for cardiac failure, whereas participants in the placebo group could have increased their risk factor for cardiac failure by as much as 4% for females and 3% for males. Therefore, over the course of one year a participant in the placebo group could increase their risk factor by as much as 14% for females and 10% for males. However, BMI is not the only metric available for predicting disease occurrence and mortality. Waist measurements are also correlated to disease occurrence^24^. Ashwell and colleagues showed the link between the waist-to-height-ratio (WHTR) is important. Participants in this study in the CFE group were able to reduce their waist circumference by 2.7 cm in 16-weeks. If maintained for 12 months, this would potentially be enough of a change the classification by an entire class (i.e. reduce classification from “action” to “take care” or from “take care” to “OK”). Together the BMI and WHTR changes seen in this study may ultimately lead to a reduced risk for disease occurrence in the CFE group and an increased risk in the placebo group.

Despite the benefits shown for food intake, satiety and body composition, the mechanism by which CFE exerts its benefits largely remains unknown. It was therefore an aim of this study to investigate possible biochemical mechanisms of CFE. Following 16-weeks of supplementation and despite the reduction in calorie intake in the CFE group, there were no associated changes in satiety hormone markers (namely leptin and ghrelin) from baseline in the CFE group. The placebo group however showed significantly elevated levels of leptin, cortisol and NPY from baseline. Typically, when leptin levels increase, satiety would improve, and weight loss or maintenance occur. However, as leptin is produced in adipose tissue^25^, as fat mass increases, leptin production increases^26,27^. Weight gain is also associated with a decreased sensitivity to leptin^27^, reducing the ability to achieve satiety. Leptin acts to reduce the desire to eat^28^, while cortisol^29^ and NPY^28^ increases the desire to eat. Therefore, at times of hunger, leptin levels are typically low and NPY levels high, initiating a feeding desire and increasing cortisol levels. The elevated cortisol leads to eating, which in turn increases leptin levels, reducing NPY activation and subsequent cortisol levels^30,31^. Cortisol can induce a dose-dependent increase in leptin^32^ and this may be why leptin and cortisol have been shown to have inverse circadian rhythms, indicating a possible satiety regulatory relationship^33,34^. This theory is partially supported by the correlation data (Table 3), where it is shown that in the placebo group, as leptin values increase so does the fat mass, while cortisol levels decrease (inverse relationship with leptin). An effect not seen in the CFE group. This is also supported by a study where CFE was shown to reduce anxiety and stress as well as act on cortisol regulation^35^.

Together, the balance of the satiety signalling molecules (leptin, NPY and cortisol) aims to keep the body in a state of homeostatic weight control. It therefore stands, that if any part of this system is compromised, weight gain, as seen in the placebo group, may occur. One possible part of the satiety pathway that can be affected is the receptor sensitivity. In the instance that sensitivity to leptin and/or NPY is affected, satiety feedback may be compromised, and regulation of satiety negatively affected. If the observed increase in leptin for the placebo group is due to reduced sensitivity, this may explain the increase in plasma NPY and cortisol in the placebo group. That is, traditionally, increased leptin signals NPY activation to stop^33,34^. However, if leptin sensitivity is reduced, even with high leptin concentrations, NPY release, and subsequently cortisol, may not be inhibited^36,37^. This combination would lead to increased food intake despite elevated leptin concentrations. Therefore, a possible mechanism by which CFE may have prevented weight gain, despite no change in satiety hormone levels, is via increased receptor sensitivity. However, the mechanism of CFE remains unclear and warrants further investigation. Future research would benefit by looking more specifically at receptor activity along the leptin-NPY satiety-signalling pathway.

Despite the best efforts to control for as many variables as possible in the current study, as with all research, there are still a number of limitations to be considered when looking at the data. The recording of some of the data (e.g. caloric intake) is primarily reliant on reporting from the participants making it open to possible errors. However, the invasive nature of many diet recording systems is either too laborious for participants to maintain and therefore increases dropout rates and/or directly influences the diet habit of an individual introducing a bias to the data. To help minimise these possible effects, all participants completed a FFQ and 24-h food recall that was matched to the diet diary data for a means to validate the data collected.

Another limitation is the use of hormone data. A consideration for use of any hormone data is the variation that can occur within any individual participant from day to day and even hour to hour. Even though time of day and activity levels were controlled for prior to any blood sampling, the participant could have experienced any number of factors prior to one blood draw and not another that could change their biochemistry profile for that day. For example, they may have woken earlier or later and/or experienced physical or emotional stress (personal or work), difficulty sleeping, poor diet and/or menstruation period that could potentially change their biochemistry profile.

The dose used may be another aspect for consideration. The present study used a dose similar to that previously shown to have efficacy. However, whether a dose that was higher (e.g. 2 × 1 g instead) would induce a greater effect is unknown. It is likely that the CFE extract has more of a chronic than acute effect, and therefore it may be beneficial to initially have a greater dose (2 g per day) and at a certain point the dose could be reduced (1 g per day). Further research is required to know what effects a different dose strategy may have.

One limitation that was difficult to avoid is the strong bias towards females in the study population. This is a result of the nature of the study. Females are far more likely to participate in a weight loss study than males are. While the subgroup analysis shows that there is no difference in the full cohort and females, the same is not able to be determined for males. Future research on CFE may be benefited by a balanced recruitment of males and females to help determine any potential difference between genders.

In summary, CFE maintained body weight, reduced waist circumference and reduced daily caloric intake over a 16-week period in overweight individuals compared to a placebo. The resulting change in calorie intake resulted in no change to satiety hormones in the CFE group. However, leptin, NPY and cortisol were all elevated in the placebo group. Future research on CFE is suggested to look at a possible link with CFE and satiety receptor sensitivity.
